# Unraveling the Regulatory Role of HuR/microRNA Axis in Colorectal Cancer Tumorigenesis

**DOI:** 10.3390/cancers16183183

**Published:** 2024-09-18

**Authors:** Vikas Yadav, Tejveer Singh, Deepika Sharma, Vivek Kumar Garg, Payel Chakraborty, Souvik Ghatak, Shakti Ranjan Satapathy

**Affiliations:** 1 Department of Translational Medicine, Clinical Research Centre, Lund University, 221 00 Malmö, Sweden; vikas.yadav@med.lu.se; 2 Translational Oncology Laboratory, Department of Zoology, Hansraj College, University of Delhi, New Delhi 110021, India; tejveer@hrc.du.ac.in (T.S.); deepika@hrc.du.ac.in (D.S.); 3 Division of Cyclotron and Radiopharmaceutical Sciences, Institute of Nuclear Medicine and Allied Sciences (INMAS-DRDO), New Delhi 110054, India; 4 Department of Medical Lab Technology, Chandigarh University, Gharuan, Mohali 140413, Punjab, India; vivek.e10915@cumail.in; 5 Amity Institute of Biotechnology, Amity University Kolkata, Kolkata 700135, West Bengal, India; pchakraborty@kol.amity.edu (P.C.); sghatak@kol.amity.edu (S.G.)

**Keywords:** microRNA, lncRNA, circular RNA, RNA-binding protein, HuR, colorectal cancer

## Abstract

**Simple Summary:**

MicroRNAs (miRNAs) play a significant role in the initiation and progression of colorectal cancer and are extensively well-studied in this context. However, the interplay between miRNAs and RNA-binding proteins, especially HuR, is yet to be fully understood. In this review, we summarize the regulatory role of the HuR-miRNA axis in colorectal cancer, highlighting its importance for an improved diagnostic or prognostic approach.

**Abstract:**

Colorectal cancer (CRC) remains a significant global health burden with high incidence and mortality. MicroRNAs (miRNAs) are small non-protein coding transcripts, conserved throughout evolution, with an important role in CRC tumorigenesis, and are either upregulated or downregulated in various cancers. RNA-binding proteins (RBPs) are known as essential regulators of miRNA activity. Human antigen R (HuR) is a prominent RBP known to drive tumorigenesis with a pivotal role in CRC. In this review, we discuss the regulatory role of the HuR/miRNA axis in CRC. Interestingly, miRNAs can directly target HuR, altering its expression and activity. However, HuR can also stabilize or degrade miRNAs, forming complex feedback loops that either activate or block CRC-associated signaling pathways. Dysregulation of the HuR/miRNA axis contributes to CRC initiation and progression. Additionally, HuR-miRNA regulation by other small non-coding RNAs, circular RNA (circRNAs), or long-non-coding RNAs (lncRNAs) is also explored here. Understanding this HuR-miRNA interplay could reveal novel biomarkers with better diagnostic or prognostic accuracy.

## 1. Introduction

Currently, cancer is one of the leading causes of death globally, and its lethality is expected to increase in the upcoming years [1]. Colorectal cancer (CRC) is the third most frequently occurring cancer worldwide, accounting for a million occurrences globally with an annual death toll of half a million [2]. For gender, it is ranked second among women and third among men, and the incidences are higher in the USA, followed by Western Europe and Australia, with a lower prevalence noted in India, China, and Africa [3].

CRC is known to be driven by inflammation, a fact supported by the efficacy of anti-inflammatory drugs in its treatment [4]. Moreover, patients with chronic inflammatory bowel diseases like ulcerative colitis (UC) or Crohn’s disease are more susceptible to developing CRC in their lifetime compared to the general population, depending on the duration and severity of the disease [5,6,7]. Inflammatory mediators produced in the arachidonic acid pathway, leukotrienes, and prostaglandins have proven impact on CRC initiation and progression [4,8]. Cysteinyl leukotriene receptor 1, prostaglandin E_2_, and cyclooxygenase-2 (COX-2) are prominent names among other mediators responsible for poor patient outcomes in CRC [8,9].

CRC originates in mucosal epithelial cells, and 70% of CRCs are sporadic without any genetic predisposition [10]. Intestinal stem cells at the bottom of the crypt undergo mutations to form micro-lesions called ‘polyps’, which further progress to adenoma and adenocarcinoma before disseminating to metastasize in distant organs like the liver or lungs [11]. Only 10% of adenomas progress to become adenocarcinoma in 12–15 years [12]. However, this can accelerate with a history of familial adenomatous polyposis (FAP) or Lynch syndrome. The most common mutation in CRC is *APC* (adenomatous polyposis coli), which accounts for 85% of total cases, and an inherited *APC* germline mutation leads to FAP. Patients lacking *APC* mutation often possess β-catenin (*CTNNB1*) mutation as a part of hyperactivated canonical WNT signaling. Other important mutations in CRC involve the activation of proto-oncogenes *KRAS* and *BRAF*, inactivation of tumor suppressors *TP53* and *SMAD4*, and alterations in *PIK3CA* (Phosphatidyl inositol 3-kinase) and *TGFBR2* (Transforming growth factor receptor-2) [10]. While the *KRAS* mutation accounts for approximately 45%, *BRAF* accounts for 5–10% of all CRC cases. The *TP53* mutation accounts for 50% of CRC cases and is associated with tumor aggressiveness and poor outcomes. Due to its slow progression from pre-malignant lesions to metastasis spreading, early detection is an integral part of an effective therapeutic regimen.

Major risk factors for CRC occurrence are divided into two categories: modifiable risk factors and non-modifiable risk factors [10]. The modifiable risk factors involve lifestyle choices, including a low-fiber diet, high consumption of red meat, alcohol, and smoking habits [13]. These risk factors are stressed by the fact that individuals who migrated from low-incidence countries to high-incidence ones may have an elevated risk of CRC. On the other hand, non-modifiable risk factors involve individual predisposition to develop CRC, where age is considered one of the major risk factors, with 30% of CRC patients diagnosed after 75–80 years of age. Although 50-year-old patients comprise only 4% of CRC patients, the incidence of CRC in younger patients from 25–35 years is on the rise [14]. Apart from age, genetic and family history could lead to FAP, or hereditary nonpolyposis colorectal cancer (HNPCC), which combined account for 20% of all CRC incidences [15,16]. Additionally, gender is associated with risk, progression, and localization in CRC. Men have a higher incidence of CRC than same-aged women, and right-sided CC is more prevalent in women, while rectal cancer is for men [17].

Screening methods like colonoscopy have improved the early detection of CRC. However, their invasive nature and high cost limit their widespread application. However, non-invasive screening tools like fecal occult blood test (FOBT) and stool DNA tests, although proven promising earlier, are unable to gather popularity due to low sensitivity yet expensive [18,19]. Hence, there is an alarming demand for new noninvasive biomarkers for both the diagnosis and prognosis of CRC.

Unlike other cancers, treatment response in CRC depends on mutation status and/or burden, which makes certain therapies effective for other solid tumors become completely ineffective for CRC patients, which poses a major bottleneck for better clinical outcomes. In CRC, conventional chemotherapy involves a combination of FOLFOX (5-fluorouracil, leucovorin, and oxaliplatin) or FOLFIRI (5-fluorouracil, leucovorin, and irinotecan), which targets the DNA damage repair pathway [20]. In addition, in metastatic CRC, anti-EGFR therapy (e.g., Cetuximab) [21] or anti-VEGFR therapy (e.g., Bevacizumab) [22] have shown promise, but immunotherapy (anti-PD1, Pembrolizumab) has only shown effectiveness in patients with high mutational burden owing to high microsatellite instability (MSI-H) [23]. In women, hormone replacement therapy (HRT) has shown a protective role against CRC. Therapeutic success in CRC is mostly context-dependent, which requires a better understanding of the mechanism of tumor progression. Additionally, biomarkers for early detection, survival prognosis, and therapy prediction are needed at this hour.

Non-coding RNAs such as microRNAs (miRNA), circular RNA (circRNA), and long non-coding RNA (lncRNA) involved in major physiological events are routinely dysregulated in cancers. Especially miRNAs are involved in CRC progression and metastasis by targeting common mutations [24,25]. Although the detailed mechanism of their action is yet to be fully understood, their role as biomarkers in various cancers has gathered attention [26]. Hence, the role of miRNAs could be explored further as a promising biomarker candidate for CRC.

RNA-binding proteins (RBPs) are known to modulate tumorigenesis by regulating the mRNA level or protein level of tumor promoters. In CRC, substantial information suggests the miRNA-mediated regulation of RBPs, an understanding of which could open new diagnostic or therapeutic avenues. In this review, we discuss the different modes of regulatory implications of miRNAs on the RBPs in CRC with a specific focus on Human antigen R (HuR).

## 2. The RBP HuR and Its Regulatory Role in CRC

RBPs play crucial roles in controlling the expression of specific genes. RBPs can create ribonucleoprotein (RNP) complexes through interactions with proteins and other types of RNAs, including mRNAs, ncRNAs, tRNAs, snRNAs, and snoRNAs [27]. RBPs influence the functions of target RNAs by recruiting different proteins and enzymes and forming distinct complexes in various combinations. In all eukaryotes, RBPs essentially affect post-transcriptional activities such as miRNA processing, mRNA splicing regulation, transport, translation, and decay [28]. RBPs are conserved in both humans and bacteria, showing their essential involvement in the cellular physiology of both species [28,29]. RBPs are affected by mutations, deletions, and/or autoimmune reactions that can change cellular functions, proper development, and a variety of illnesses. Changes in the expression of RBPs are frequently observed in a range of neurological conditions, including autoimmunity and cancer. RBP’s subcellular localization and associated RNA substrates influence their regulatory roles as well since post-transcriptional events are frequently performed in subcellular compartments that are divided by phases and membranes [30,31]. Improved targeted therapeutics may result from a greater understanding of the molecular mechanisms underlying aberrations of RBPs in several cancers, including CRC [30,31,32]. By establishing feed-forward and feedback loops, RBPs offer a way to combine the many gene expression processes and estimate the length of time a system will react to a disturbance. The scientific community believes that RBPs’ functions in producing and preserving RNA will grow along with the transcriptome’s diversity.

HuR, a prominent RBP, is one of the key regulators of cellular proliferation, which belongs to the Hu protein family and the Embryonic Lethal Abnormal Vision in Drosophila (ELAV) family [33]. The earliest evidence that ELAV/Hu proteins might play a role in growth regulation came from their identification as specific tumor antigens in the tumors of individuals with paraneoplastic neurological disorders [34]. It was later discovered that ELAV/Hu proteins, which include HuB, HuC, and HuD (mainly expressed in neuronal tissues), and the ubiquitous HuR, regulate the expression of labile mRNAs with AU- and U-rich sequences by improving their translation, stability, or both [35]. Many of these target mRNAs encode proteins crucial for cell division and growth.

HuR is a key protein in tumorigenesis [36], primarily localized in the nucleus, and can shuttle between the nucleus and cytoplasm in intestinal epithelial cells [37]. The stabilization of HuR-targets often results in increased mRNA stability and enhanced protein expression of HuR target genes involved in cell proliferation, apoptosis resistance, and metastasis [38,39].

**Figure 1:**
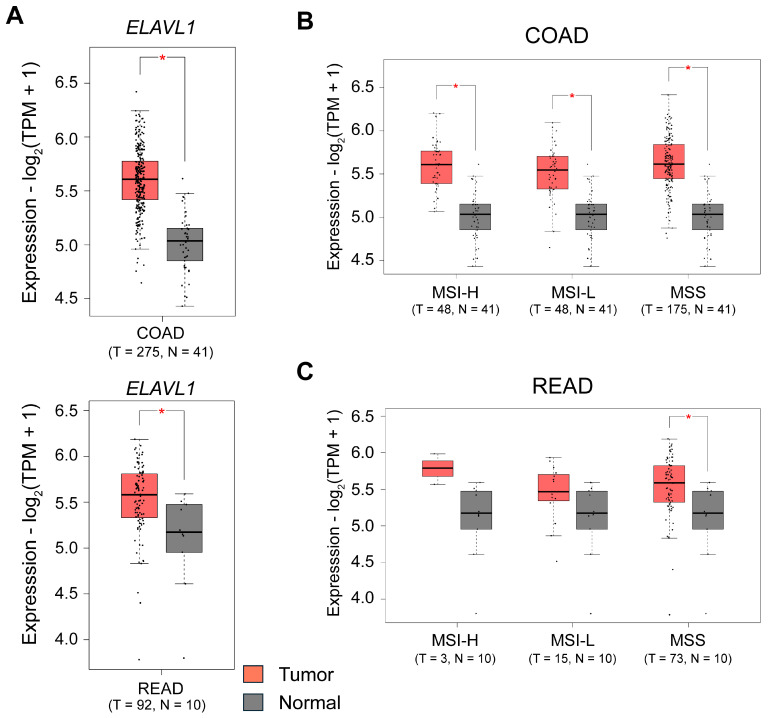
Expression of HuR in colon and rectal adenocarcinoma patients. Box plots showing expression of (**A**), *ELAVL1* (gene encoding human antigen R; HuR) in colon adenocarcinoma (COAD) and rectal adenocarcinoma (READ) patients in The Cancer Genome Atlas (TCGA) COAD and READ cohorts generated using the Gene Expression Profiling Interactive Analysis 2 (GEPIA2) http://gepia2.cancer-pku.cn/) [48]. Box plots showing expression of *ELAVL1* expression in (**B**), TCGA-COAD and (**C**), TCGA-READ cohorts in patients grouped based on the microsatellite stability status into microsatellite instability-high or low (MSI-h, MSI-l) or microsatellite stable (MSS). Comparisons are performed between cancer tissue and normal tissue. The number of patients (*n*) is indicated in the figure. The log2 fold change and *p*-value cut-offs were according to the default settings of GEPIA2. T = Tumor, N = Normal.

It is overexpressed in colorectal adenocarcinomas and binds to targets via AU-rich element (ARE) motifs, promoting increased mRNA stability (Figure 1) [40,41]. The functions of HuR in CRC pathogenesis have been well-studied. According to reports, HuR is increased in CRC [42,43,44,45]. It stabilizes several oncogenes, including COX-2 [43,45], VEGF [46], and IL-8 [46], which promotes the proliferation and tumorigenicity of CRC cells. Increased cytoplasmic HuR levels were reported to be strongly correlated with COX-2 expression and the stage of CC [43,45]. HuR markedly stimulates the formation of xenografted tumors in a mouse model of CRC [42].

In the last few years, evidence has shown that HuR’s influence on bound transcripts depends on its interaction with miRNAs, and competition between HuR and miRNAs enhances target gene expression, while their cooperation results in reduced expression [47]. MiRNAs are among the few known regulators of HuR expression levels and, hence, passively affect certain biological processes.

## 3. Intriguing Role of miRNAs-HuR Axis in CRC

### 3.1. MiRNAs and Their Role in Cancer Pathogenesis

MiRNAs are so far the most studied type of small noncoding RNA molecules, typically 20–25 nucleotides long, that play a vital role in silencing target genes and regulating post-transcriptional processes [49,50]. They bind to specific mRNA molecules and inhibit protein production by promoting mRNA degradation or reducing translation efficiency. A single miRNA can target multiple mRNAs, and various miRNAs can act on the same mRNA as well [50,51,52,53]. Several computational tools have been developed to predict miRNA targets, which are then experimentally validated [54,55,56]. MiRNAs regulate nearly two-thirds of human protein-coding genes, influencing key cellular processes like migration, proliferation, differentiation, cell death, and metabolism [57,58]. However, because miRNA-mediated dynamics in biological networks are different, it is difficult to explain how miRNAs organize biological decision-making events [59].

Trieber et al. and several others have extensively discussed the biogenesis of miRNA before; therefore, we have not discussed it here in detail [60]. In miRNA biogenesis, a pri-miRNA (capped at the 5’ end and polyadenylated at the 3’ end) is transcribed by RNA Pol II. Endonucleases like DROSHA and DGCR8 process this precursor in the nucleus, producing an approximately 85-nucleotide stem-loop structure known as pre-miRNA [61,62]. Exportin-5 then transports the pre-miRNA to the cytoplasm, where the ribonuclease Dicer cleaves it into a double-stranded mature miRNA (22–30 nucleotides). The mature miRNA duplex associates with Argonaute proteins to form the RNA-induced silencing complex (RISC), which regulates translation by binding to complementary sequences in the 3’ UTR of target mRNAs via the seed region (2–8 nucleotides) [63,64]. Through these interactions, miRNAs form imperfect hybrids with target mRNAs, altering their stability and reducing the expression of around 30% of protein-coding genes [65].

Mutations or abnormal miRNA expression can contribute to cancer development and progression. Depending on the cellular context, miRNAs can function as either oncogenes or tumor suppressors [64,66]. Abnormal miRNA expression may result from gene amplification, deletion, or translocation of genomic regions containing miRNA genes. Additionally, miRNA genes can undergo epigenetic modifications, promoting oncogenesis in various cancers. These epigenetically altered miRNA genes are considered potential biomarkers for cancer diagnosis and monitoring disease progression [67,68].

**Figure 2:**
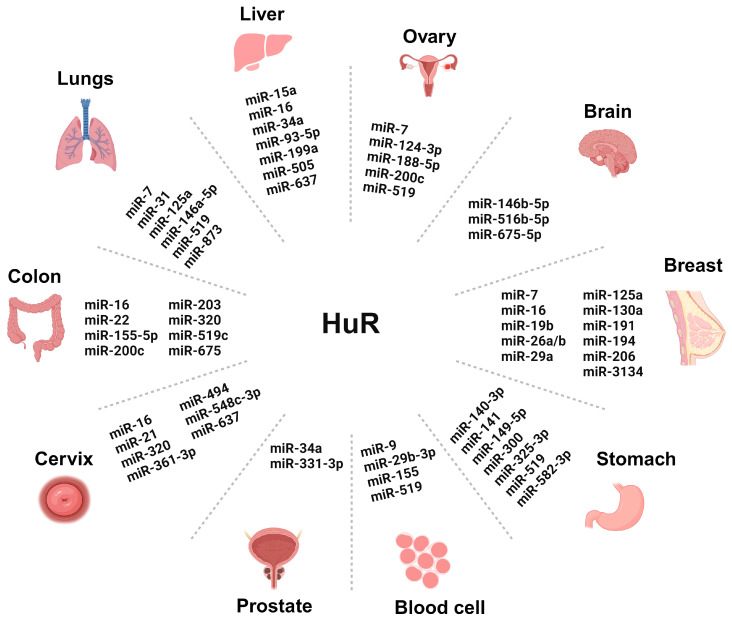
Graphics showing the role of different microRNAs (miRNAs) regulating human antigen R (HuR) in cancers of various organs. The names of the miRNAs are mentioned in the graphics. The miRNAs involved in gallbladder cancer (miR-502-3p), thyroid cancer (mir-31 and miR-19), sarcoma (mir-29), bladder cancer (miR-494), and kidney cancer (miR-519) are not mentioned in the graphics due to space restrictions. Each mentioned miRNA is either a tumor promoter or tumor suppressor in the specific cancer. The image was created with the help of Biorender.com.

More than 52% of miRNA genes associated with cancer suppression are located in fragile regions, which may lead to malfunction and abnormal miRNA expression in different types of cancer. These small molecules are crucial in regulating various cellular and biological processes, such as inflammation, stress response, migration, differentiation, apoptosis, metabolism, extracellular matrix regulation, MET transcription factors, and cancer initiation and progression. RBPs are also known to regulate miRNAs in several cancers [69]. They also impact tumor survival through pathophysiologic mechanisms like chemoresistance, chemosensitivity, and radiation resistance [70]. RBP HuR is reported to be associated with numerous miRNAs in different cancers (Figure 2) [47,71]. Oncogenic miRNAs, also known as oncomiRs, target and inhibit endogenous tumor suppressor genes, accelerating carcinogenesis [72].

#### 3.1.1. Role of oncomiRs in HuR Regulation

Upregulation of oncomiRs is significantly known to impact the progression of CRC (Table 1). MiR-21 is a highly upregulated gene in CRC that targets and downregulates genes like *PDCD4*, *TIAM1*, *SPRY2*, *PTEN*, *TGFBR2*, and *CDC25A* [70,73]. It functions as an oncomiR, downregulating *PTEN* expression in CRC cells. MiR-21’s high expression is associated with resistance to chemotherapy of 5-FU, and its expression directly targets the 3’-UTR of tumor suppressor human DNA MutS homolog 2 (hMSH2), reducing 5-FU-induced damage arrest and apoptosis [74,75]. Overexpression of miR-21 enhances cell proliferation and invasion, inhibits apoptosis against 5-FU treatment, and vice versa [76]. These findings suggest miR-21 could be a non-invasive biomarker for CRC diagnosis and prognosis.

MiR-92a is a member of the oncomiR complex miR-17-92, which plays a key role in tumor cell proliferation, angiogenesis, and tumor progression in various cancers [77]. It is overexpressed in CRC, leading to suppression of E-cadherin and activation of β-catenin and vimentin, which promote EMT via the PTEN/PI3K/AKT pathway [78]. Elevated miR-92a, as shown by Nishida et al., is linked to lymphatic and venous invasion as well as liver metastasis through the TGF-β pathway [79]. CRC patients with high miR-92a expression have poor overall survival compared to those with low miR-92a expression [80]. Additionally, increased levels of miR-92a in CRC patients’ body fluids (e.g., plasma) have shown specificity and could be used for identifying individuals with CRC. This makes miR-92a a possible prognostic and diagnostic biomarker candidate for CRC [81].

MiR-96 is also upregulated in CRC patients and is linked to liver metastasis. It drives cell growth and proliferation by targeting TP53INP1, resulting in the downregulation of p53, FOXO1, and FOXO3a [82]. MiR-96 further enhances the effectiveness of 5-FU by inhibiting XIAP and UBE2N, leading to apoptosis [83]. Moreover, it also increases chemosensitivity to the platinum-based drug cisplatin and the PARP inhibitor AZD2281 by suppressing RAD51 recombinase and REV1 DNA polymerase, as evident in both in vitro and in vivo [83].

The miR-135 family has two isoforms: miR-135a and miR-135b. Both isoforms are conserved in mammals [84] and are considered oncogenic miRNAs in CRC, with miR-135b being overexpressed in cancer tissue than in normal tissue. Overexpression of miR-135 leads to the downregulation of *APC*, an essential component of the degradation complex in the Wnt signaling pathway. Furthermore, miR-135 serves as a biomarker for assessing the clinical stage of CRC [85,86].

**Table 1:** Role and function of miRNAs in CRC. MiRNAs, with their function as either oncomiR or tumor suppressor miR, are mentioned here.

No.	microRNA	Function	Reference
1.	miR-21	OncomiRs	[74]
2.	miR-92a	OncomiRs	[78]
3.	miR-96	OncomiRs	[82]
4.	miR-135	OncomiRs	[86]
5.	miR-16	Tumor suppressor miR	[87]
6.	miR-22	Tumor suppressor miR	[88]
7.	miR-34	Tumor suppressor miR	[89,90]
8.	miR-194	Tumor suppressor miR	[91,92,93,94]
9.	miR-143	Tumor suppressor miR	[95,96]
10.	miR-145	Tumor suppressor miR	[96]
11.	miR-155-5p	Tumor suppressor miR	[97]
12.	miR-519c	Tumor suppressor miR	[98,99]
13.	miR-324-5p	Tumor suppressor miR	[100,101]

#### 3.1.2. Role of Tumor Suppressor miRs in HuR Regulation

MiRNAs also play a role as tumor suppressors by downregulating oncogenes involved in proliferation, apoptosis, invasion, and migration [72]. The miR-34 family is a prominent tumor suppressor miRNA due to its combinatorial effect with the tumor suppressor gene *TP53*.

It comprises three members, miR-34a, miR-34b, and miR-34c, located in the long arm 2 region of chromosome 11 [102]. MiR-34a, a tumor suppressor, regulates markers in cell proliferation, apoptosis, and senescence [103,104]. In cancer, miR-34a is downregulated, while HIF1 promotes angiogenesis by releasing VEGF from miRNA-mediated downregulation [103,104]. MiRNAs predominantly regulate target gene expression through a negative feedback loop or an incoherent feedforward loop [105]. MiR-34b-5p, another member of the miR-34 family, plays a crucial role in multiple diseases, including cancer. In CRC, long noncoding RNA (lncRNA) LINC02418 with a binding sequence for miR-34b-5p negatively affects patient survival, possibly via the miR-34b-5p/Bcl-2 axis.

The Lethal-7 (let-7) family, comprising 12 miRNAs, is essential in the epithelial-mesenchymal transition (EMT) process by targeting oncogenes like RAS, c-Myc, CDC25A, CDK6, and HMGA2. In CC tissues, let-7 is downregulated compared to adjacent noncancerous tissues, and high expression of let-7 is significantly correlated with better survival outcomes in CRC patients [106,107,108,109].

Additionally, miR-194 is mostly suppressed in CRC and is closely linked to overall survival and tumor size. Although it acts as a tumor suppressor in CRC, it is not associated with lymphatic invasion or distant metastasis. The expression profiles of the miR-194 target genes are in line with the oncogenic transcriptional regulator *HMGA2*, which is involved in restricting cell proliferation and migration, decreasing tumor growth in vivo, attenuating cell migration, suppressing EMT, and improving anticancer drug sensitivity in CRC [91,92,93,94].

MiR-143 and miR-145, located in a 1.6 kb cluster on the 5q32-33 chromosomal region, are frequently co-expressed from the same promoter. They are downregulated in several cancer types, including CRC, and function as anti-tumorigenic agents by altering various cancer-related cellular processes. The pathophysiology of synchronous CRC may be functionally associated with the deregulation of the miR-143-145 cluster [95,96].

Abdelmohsen et al. reported that miR-519 downregulated HuR expression in human CC cell lines RKO and HCT116 by suppressing HuR translation without affecting HuR mRNA stability [98]. HuR translation was more strongly suppressed by the coding region (CR) location of miR-519 interaction on HuR mRNA. When miR-519 levels increased, HuR levels decreased, suggesting an inverse correlation between the two. Normal tissues expressed low HuR and high miR-519 levels, but malignant tissues in cancer patients expressed high HuR and low miR-519 levels [98,99]. MiR-519’s potential as a tumor suppressor miRNA is also suggested by the fact that overexpression of the miR-519 reduced carcinogenesis by inhibiting DNA replication and cell division [99].

Using CRC patient tissue, Liu et al. have shown that tumor suppressor miR-22 was lowly expressed in CRC tissue compared to the normal tissue [88]. Expression of miR-22 was inversely correlated with HuR expression, and patients with high expression of miR-22 have a better prognosis. Furthermore, miR-22 directly interacts with the 3’-UTR of HuR, which inhibits HuR and hence limits the proliferation and migration of CRC cells in vitro.

Gu et al. have reported that MiR-324-5p is downregulated in CC cells (SW480 and SW620), while HuR (ELAVL1) expression is elevated in the same cell lines [100]. Cells treated with miR-324-5p mimics could downregulate the expression of ELAVL1 in both CC cells with a decrease in proliferation. Activation of miR-324-5p further limited the metastatic ability of CC cells by decreasing migration and invasion via targeting MMP-9 and the urokinase-type plasminogen activator system (uPA).

The inflammatory mediator, COX-2, produced in the arachidonic acid pathway, is well known as a tumor promoter in CRC [4]. HuR interacts with miR-16 to promote COX-2 mRNA stability and elevate its expression [87]. Due to this interaction, the steady-state levels of miR-16 decline rapidly. Cytoplasmic accumulation of HuR also leads to HuR binding to miR-16, causing a decrease in COX-2 abundance [87]. Although the molecular mechanism behind this reduction is unknown, these findings suggest that HuR could upregulate COX-2 expression by directly binding to COX-2 3’UTR and indirectly reducing miR-16 abundance. Additionally, antagomiR-155-5p reduces cytoplasmic HuR expression, facilitating HuR-mediated impact on the pro-inflammatory and pro-oncogenic mRNAs. Co-transfection with a blocker targeting TS1 ARE motifs reverses the reduction of HuR protein, suggesting that miR-155-5p affects HuR levels through mRNA expression rather than nucleus and cytoplasm reorganization [97].

Taken together, it is evident that a complex interplay between miRNAs and HuR possibly regulates tumor-promoting or tumor-suppressing functions. However, the HuR-miRNA axis is also known to be regulated by another type of non-coding RNA, circRNAs.

## 4. CircRNA Mediated Modulation of miRNAs-HuR Axis in CRC

**Figure 3:**
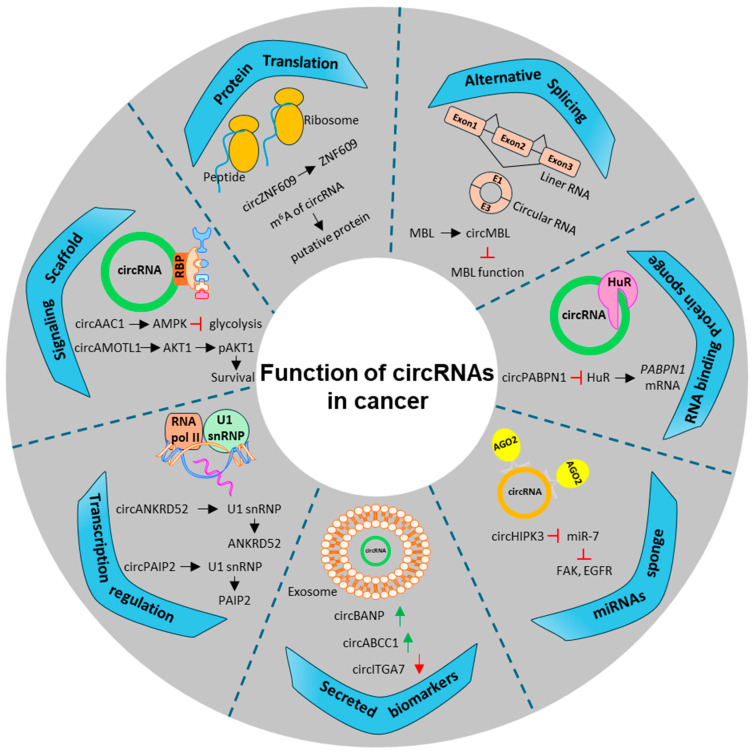
Schematics show the different functions of circular RNAs (circRNAs) involved in cancer. Briefly, the image shows how circRNAs are categorized and presented based on their function, ranging from transcriptional to translational regulation, miRNA sponging, alternative splicing, and RBP sponging. We have also shown examples of circRNA that are predicted to be probable biomarkers and signaling scaffolds. The black arrow shows the signaling pathway. The red arrow shows downregulation, and the green arrow shows upregulation. The other colors used in the graphics are only for better illustration and do not represent explicitly anything.

CircRNAs have recently gained significant attention for their diverse roles in gene regulation. Unlike linear RNA, circRNAs form continuous loops through covalent bonds generated by the back-splicing mechanism, making them more stable and resistant to degradation by RNA exonucleases [110]. Reports suggest that circRNAs can act as oncogenes or tumor suppressors by serving as miRNA sponges, binding to RBPs, regulating alternative splicing and transcription, encoding peptides, regulating host gene expression, or functioning as exosomal circRNAs (Figure 3) [111,112,113]. Additionally, circRNA concentrations, isoform expressions, and types are cell-type and tissue-specific [114,115,116,117], and they can be detected in human body fluids (e.g., saliva or plasma), which makes circRNAs and their host genes bona fide candidate biomarkers for early diagnosis, disease prognosis, and effectiveness of treatment for various tumors [118,119]. Although the biogenesis and function of circRNAs were previously poorly understood, with advances in sequencing techniques and bioinformatics analyses, researchers have better understood their roles and mechanisms.

### 4.1. CircRNA and Their Role in Gene Regulation

CircRNAs influence host gene transcription by interacting with RNA Pol II, recruiting various proteins, or forming R-loops to target regions regulating transcription [114,120,121,122,123,124,125]. Zhang et al. have shown that circANKRD52, circMCM5, and circSIRT7 accumulate at the transcription sites of their respective host genes, facilitating RNA Pol II-mediated transcription elongation and serving as positive regulators of transcription [114]. Moreover, reports show that circEIF3J and circPAIP2 bind to RNA Pol II, U1 snRNP, and promoters of their host genes, further amplifying transcription through a cis-acting positive feedback loop. Silencing these circRNAs results in reduced transcription levels of EIF3J and PAIP2 [120].

When RNA polymerase is silenced or RNA biogenesis is disrupted, R-loop structures composed of RNA-DNA hybrids and displaced single-stranded DNA can form [126,127], potentially interfering with replication and transcription processes. Recent studies indicate that circRNAs can create DNA hybrids or R-loops, increasing the efficiency of exon-deficient mRNA cleavage. Beyond altering linear transcript levels, circRNAs can trap mRNA, halt transcription, and enhance splicing factors, thereby regulating host gene expression [123,125]. Xu et al. identified that circSMARCA5 forms an R-loop to regulate its host gene expression [124]. Additionally, circRNAs are known to contain specific RNA-binding protein sites, which allow them to act as either protein decoys or scaffolds and guide proteins to certain promoter regions. This enables circRNAs to regulate transcription activation and host gene expression, potentially contributing to tumor progression. The following section will broadly discuss it, focusing on post-translational regulation by miRNA and protein sponges.

### 4.2. Regulatory Role of circRNA in the Translational and Post-Translational Modification of Their Host Genes

CircRNAs regulate the host gene translation by binding to the specific translation initiation-related proteins, influencing protein synthesis, and contributing to tumor development or progression. Yes-associated protein (YAP), a crucial factor in the Hippo pathway, is often dysregulated in CRC apart from other cancers [128]. CircYAP binds to *YAP* miRNA, translation initiation proteins eIF4G, and poly (A) binding protein (PABP), disrupting the interaction between eIF4G on the 5’-cap and PABP on the poly (A) tail of the *YAP* mRNA translation complex. This indicates that circYAP could act as a tumor suppressor gene, which could be validated with further investigations [129].

Furthermore, circRNAs can also serve as translation activators or inhibitors by influencing the binding of RBPs to host gene mRNA. For example, circPABPN1, derived from *PABPN1*, can compete with HuR by regulating *PABPN1* [128,130] CircRNAs are also known to directly interact with host proteins to regulate post-translational modification, an essential process for maintaining protein turnover [131,132,133].

### 4.3. CircRNA as miRNA Sponges, Decoys, or Scaffolds

In addition to their above-mentioned role, circRNAs can also act as miRNA sponges or competing endogenous RNAs (ceRNAs) [134]. They contain miRNA response elements (MREs), which bind to and isolate miRNAs, preventing them from interacting with their target mRNAs. By sponging and binding to miRNAs, circRNAs block miRNAs from inhibiting their target genes. This leads to the upregulation of these target genes along with the protein-coding genes, which further regulate specific signaling cascades [135,136,137]. Moreover, some circRNAs are reported to act as ceRNAs in order to regulate the expression of other target genes and contribute to tumor development [137], like circEZH2/miR-133b/IGF2BP2/CREB1 [138]. CircCDR1as (ciRS-7) contains over 70 conserved miR-7 binding sites. CDR1as acts as a sponge by binding to miR-7 and regulating miR-7 target genes [139]. Circular RNAs may compete with non-coding RNAs or proteins for binding to regulatory molecules such as proteins RBPs during decoy activities [140]. Competition alters the availability and function of regulatory molecules, thus affecting gene expression. CircRNAs act as miRNA sponges, decoys, or scaffolds through distinct mechanisms by interacting and binding with regulatory molecules such as miRNAs and proteins.

In CRC, Tan et al. have reported that circRNA circ0104103 is downregulated in CRC tissue, and its activation leads to inhibition of cell proliferation, migration, and invasion, as evident using in vitro and in vivo models of CRC [141]. Circ0104103 acts as a ceRNA by negatively regulating endogenous miRNA-373-5P as a sponge, hence depleting the expression of HuR. Using both overexpression and silencing of circ0104103, authors have proved its tumor suppressor role in CRC cells.

Additionally, circAGO2 is over-expressed in CRC cells and directly interacts with HuR [142]. It induces the HuR translocation from nuclear to cytoplasm, further activating HuR protein and its associated miRNAs. The CircAGO2/HuR complex regulates cancer-promoting genes, like *HNF4*, *NOTCH4*, and *SLC2A4*, by regulating the miRNAs, miR-224-5p and miR-143-3p, located close to the ARE of these oncogenes. Anti-cancer therapies targeting the circAGO2/HuR complex could be effective in CC.

CircRNA can act as a protein sponge or decoy by binding to a specific region of the target promoter, regulating the activity, localization, or stability of the target proteins. RBPs, DNA demethylases, and DNA methyltransferases play crucial roles in this type of interaction. As a result, transcription, splicing, or other regulatory processes will be altered. CircFoxo3, a circRNA derived from the *FOXO3* gene, binds to various proteins involved in cell cycle regulation, such as CDK2 and p21 [143,144], which in turn not only regulates their activity and cellular localization but also influences cell cycle progression. It has been demonstrated that some circRNAs transcriptionally activate host genes and their downstream target genes involved in either tumor promotion or inhibition [122,145].

### 4.4. Role of circRNA in the Regulation of miRNA-HuR Axis in CRC

CircRNAs regulate the miRNA-HuR axis, which contributes significantly to cancer progression and metastasis in CRC [146,147]. CircRNAs can serve as sponges for miRNAs that target HuR mRNA (Table 2). As circRNAs sequester these miRNAs, they prevent them from binding to and downregulating HuR mRNA. Through this interaction, HuR mRNA is stabilized and expressed more, which results in a greater level of HuR protein and subsequent effects on gene expression in CRC cells [142]. Among the well-studied circRNAs in CRC is circHIPK3, which acts as a sponge for miRNAs targeting HuR mRNAs, including miR-7, miR-124, and miR-29b [148,149]. As circHIPK3 binds to these miRNAs, it derepresses HuR expression, promoting CRC progression by inducing HuR-mediated proliferation and metastasis [150].

CircRNA microarray analysis identified that the two anti-metastatic circRNAs, circPPFIA1-L and circPPFIA1-s, are significantly downregulated in CRC cells [151]. CircPPFIA1-L and circPPFIA1-s acted as sponges to the oncomiR miR-155-5p. While circPPFIA1-s upregulated tumor suppressor CDX1 by decoying miR-155-5p; it downregulated oncogene RAB36 by disrupting the miR-155-5p/HuR complex and separating HuR. CircNOLC1 plays a vital role in CC liver metastasis [152]. CircNOLC1 upregulates the *NOLC1* mRNA expression via HuR. It also sponges miR-212-5p to upregulate c-Met in liver metastasis.

**Table 2:** Role of circRNA/lncRNA in miRNA-HuR axis regulation in CRC.

No.	microRNA (Expression)	Regulation by Other Non-Coding RNA	Reference
1.	miR-155-5p	circPPFIA1-L, circPPFIA1-S (circRNA)	[151]
2.	miR-373-5p	circ0104103 (circRNA)	[141]
3.	miR-143-3p miR-224-5p	circAGO2 (circRNA)	[142]
4.	miR-212-5p	circNOLC1 (circRNA)	[152]
5.	miR-34b-5p	OIP5-AS1 (lncRNA)	[153]
6.	miR-3121-3p	TNFRSF10A-AS1 (lncRNA)	[154]

CircPABPN1 and circRHOBTB3 have also been shown to promote CRC cell proliferation and migration by sponging miR-675, which targets HuR mRNA [155,156]. As a result of this circRNA-mediated regulation, HuR protein levels are upregulated, which facilitates tumor progression. CRC cell survival, proliferation, and metastasis are promoted by circRNA-mediated regulation of the miRNA-HuR axis. CRC progression may be associated with HuR’s ability to stabilize mRNAs encoding proteins involved in the cell cycle (e.g., cyclins, CDKs, and apoptosis resistance proteins, such as the Bcl-2 family) and epithelial-mesenchymal transition (EMT) genes. Circular RNAs that regulate the miRNA-HuR axis could potentially be targeted as therapeutics for CRC. The use of antisense oligonucleotides (ASOs) or small molecule inhibitors to inhibit or disrupt specific circRNA-miRNA interactions may offer novel approaches to treating CRCs. A circRNA-miRNA interaction affects the miRNA-HuR axis, modulating HuR expression and downstream gene targets in CRC. A better understanding of the miRNA-HuR axis provides insight into potential therapeutic strategies to intervene in the pathogenesis of CRCs by targeting the miRNA-HuR axis.

## 5. Intriguing Role of lncRNA in the Regulation of miRNA-HuR Axis in CRC

The last type of non-coding RNA we discuss here as a regulator of the HuR-miRNA axis is the lncRNAs. lncRNAs are produced from widespread genome transcription. These RNAs are longer than 200 nucleotides and cannot translate into functional proteins. This broad term comprises an enormous and tremendously varied group of transcripts with several genetic origins and biogenesis. The human genome has more than 16,000 lncRNA genes, which is shown by Human GENCODE Statistics, though a few other databases show that humans have more than 400,000 lncRNAs [157,158]. These RNAs control gene expression at various stages, including RNA splicing, chromatin structure, function, transcription of neighboring and distant genes, translation, and stability. Chromatin relaxation could happen when positively charged histone tails neutralize the negative charge of RNA molecules, which suggests that RNA-mediated chromatin relaxation could be an unusually fast event that leads to specific gene activation [159]. Numerous lncRNAs on chromatin have been revealed to interact with proteins, thus either upregulating or downregulating their binding and activity at specific sites of DNA. Additionally, direct lncRNA transcriptional effects on the gene of interest can be enabled by protein-assisted long-range chromatin interactions, like those facilitated by CTCF, a zinc finger protein [160,161,162]. About 20% of all lncRNAs in mammalian genomes are divergent lncRNAs transcribed in the opposite direction from neighboring protein-coding genes. Luo et al., through a genome-wide study, identified that there is a high correlation between the distribution of this class of lncRNAs and critical developmental regulating genes [163]. Additionally, lncRNAs are involved in the development and regulation of nuclear condensates and organelles [164]. Studies have shown that membrane-free RNA-protein compartments called nuclear condensates are essential for several biological activities [165]. There is a close relationship between the spatial regulation of gene expression during development and the abundance of lncRNAs associated with chromatin-modifying complexes, transcribed from enhancers, and nucleate phase separation of nuclear condensates and domains [166]. Using super-resolution microscopy, West and colleagues identified nuclear structures known as paraspeckles, constructed around the lncRNA Neat1. Neat1 controls several physiological functions, such as the development of the corpus luteum and the progression of cancer [167]. Recently, several authors have reviewed the detailed transcriptional regulatory activity of lncRNAs in the context of development and disease biology [164,166,168].

Dysregulated expression of lncRNAs and miRNAs has been identified as a measure of independent cancer patient prognosis and a characteristic of cancer progression [169,170]. Several studies have highlighted a strong relationship between miRNA and lncRNA in regulating the transcriptional regulation of RNA [169,171]. By sequestering miRNAs, lncRNAs can function as miRNA decoys, competing with endogenous RNAs and causing miRNA target genes to re-express. Furthermore, lncRNAs can enhance gene expression by competing with miRNAs for specific binding sites in the non-coding sections of mRNAs, thus blocking the transcriptional repression caused by miRNAs. The strong interaction between several kinds of non-coding RNA shows that some lncRNAs can be processed into miRNAs [169,172,173]. There is growing evidence that lncRNAs are important for the initiation and development of CC [174,175,176]. Forrest et al. used RNA-sequencing data from the TCGA dataset to find approximately 200 lncRNAs that were differentially expressed in colon tumors [174]. Kasagi and colleagues in their study found Colon-cancer Associated Transcript 2 (CCAT2), a substantially overexpressed lncRNA in microsatellite-stable CRC, to circumscribe a single nucleotide polymorphism (SNP) rs6983267, which is a high-risk allele for CRC [177]. In a separate study, Ling et al. demonstrated that CCAT2 transduced HCT116 cells exhibit enhanced migration capacity, and highly metastatic KM12SM silenced for CCAT2 exhibits diminished invasion rates [178]. The authors further found that CCAT2 physically interacts with TCFL2 and MYC-regulated miRNAs miR-17-5p and miR-20a. Notably, miR-20a was overexpressed during the change from colon mucosa to early adenoma and is expected to target several genes involved in the WNT pathway [178]. Alshahrani et al. and Rajtmajerova et al. have extensively reviewed more examples where the association between the lncRNA-miRNA axis and CRC development is described in detail [179,180].

To explore the regulatory mechanism of the lncRNA-miRNA axis in CRC development, Wang et al. found OIP5-AS1 lncRNA to positively interact between the HuR protein and miR-34b-5p [153]. Furthermore, they explored the carcinogenic potential of OIP5-AS1, revealing its overexpression in CRC patients along with HuR. The simultaneous inhibition of HuR reduces the levels of OIP5-AS1, leading to a concomitant decrease in CRC growth. Mechanistically, authors found that HuR binds to and stabilizes OIP5-AS1 expression in CRC cells, whereas miR-34b-5p competes with HuR in binding to OIP5-AS1 [153]. Similarly, Wang et al. revealed the carcinogenic potential of TNFRSF10A-AS1 in CRC progression, revealing its overexpression in CRC patients along with HuR [154]. Through in vitro luciferase assay and in silico analysis, the direct interaction between TNFRSF10A-AS1 and miR-3121-3p was verified. However, miR-3121-3p was found to be downregulated in CRC patients, suggesting a negative correlation with TNFRSF10A-AS1. Hence, the authors proposed that the oncogenic activity of TNFRSF10A-AS1 was via sponging miR-3121-3p. They showed that TNFRSF10A-AS1 binds to miR-3121-3p, thereby reducing the suppressing effect of miR-3121-3p on its downstream target, HuR. Conclusively, both studies highlighted contrasting but crucial insights on how the lncRNA/miRNA/HuR axis plays a significant role in the progression of CRC, thereby providing a platform for the diagnosis and treatment of CRC.

## 6. Conclusions and Future Directions

The HuR-miRNA interplay is complex, particularly in CRC. MiRNAs regulate HuR levels, while HuR, in turn, regulates the levels of miRNAs. The role of this ironic relationship between HuR-miRNA and its impact on mRNA translation in CRC remains poorly understood and requires further investigation [181]. A deeper understanding of the precise binding sites of HuR and miRNAs, their spatial arrangement, and how their interactions influence the secondary structure of the RNA is essential. Such studies would help understand how these interactions influence mRNA stability and translation in CRC. Hence, investigations focused on individual mRNAs are required [182,183]. While competitive regulation of target mRNA degradation by HuR and miRNAs has been observed, high-throughput analyses have not yet revealed more widespread instances of this process. To gain further insights, it would be beneficial to use experimental approaches such as identifying Ago2 binding sites under conditions of cellular stress, mitogenic signaling, or in the presence or absence of HuR [184]. Moreover, high-throughput techniques like polysome profiling, nascent translation assays, and pulsed SILAC should be used to investigate alterations in translation. This approach would help distinguish between direct miRNA-mediated regulations of HuR abundance and HuR-mediated regulation of miRNA levels, a distinction critical for developing novel biomarkers or therapeutic regimens for CRC [33,185]. Therapeutically, targeting the HuR-miRNA axis presents a promising avenue for CRC treatment. By manipulating this regulatory network, it may be possible to affect mRNA translation, which would either help suppress tumor growth or enhance the efficacy of existing treatments. Small molecules or antisense oligonucleotides designed to disrupt specific HuR-oncomiRs interactions could reduce HuR’s stabilizing effect on oncogenic mRNAs, thereby minimizing cancer progression. Additionally, therapies that modulate miRNA levels to alter HuR activity could benefit precision therapy against CRC. Although few preliminary studies discussed in the review article suggest intricate relationships of the HuR-miRNA axis in CRC progression, we believe that more studies are needed to strengthen such claims for diagnostic or therapeutic purposes in the future. Thus, advancing our understanding of the HuR-miRNA relationship will be crucial for developing biomarkers and targeted therapies and improving clinical outcomes in CRC.
